# Activation of Cannabinoid Receptor 2 Enhances Osteogenic Differentiation of Bone Marrow Derived Mesenchymal Stem Cells

**DOI:** 10.1155/2015/874982

**Published:** 2015-01-21

**Authors:** Yong-Xin Sun, Ai-Hua Xu, Yang Yang, Jia-Xing Zhang, Ai-Wen Yu

**Affiliations:** Department of Rehabilitation, The First Affiliated Hospital, China Medical University, Huaxiang Road No. 39, Tiexi District, Shenyang 110001, China

## Abstract

Bone marrow derived mesenchymal stem cells (BM-MSCs) are considered as the most promising cells source for bone engineering. Cannabinoid (CB) receptors play important roles in bone mass turnover. The aim of this study is to test if activation of CB_2_ receptor by chemical agonist could enhance the osteogenic differentiation and mineralization in bone BM-MSCs. Alkaline phosphatase (ALP) activity staining and real time PCR were performed to test the osteogenic differentiation. Alizarin red staining was carried out to examine the mineralization. Small interference RNA (siRNA) was used to study the role of CB_2_ receptor in osteogenic differentiation. Results showed activation of CB_2_ receptor increased ALP activity, promoted expression of osteogenic genes, and enhanced deposition of calcium in extracellular matrix. Knockdown of CB_2_ receptor by siRNA inhibited ALP activity and mineralization. Results of immunofluorescent staining showed that phosphorylation of p38 MAP kinase is reduced by knocking down of CB_2_ receptor. Finally, bone marrow samples demonstrated that expression of CB_2_ receptor is much lower in osteoporotic patients than in healthy donors. Taken together, data from this study suggested that activation of CB_2_ receptor plays important role in osteogenic differentiation of BM-MSCs. Lack of CB_2_ receptor may be related to osteoporosis.

## 1. Introduction

Bone tissue engineering provides alternative methods for bone defect treatment besides traditional solutions used in clinics, including autologous and allogeneic bone graft, vascularized grafts of the fibula and iliac crest, and other bone transplantation techniques [1]. Bone tissue engineering constructs may potentially show better mechanical features than bone grafts [2]. It may be very helpful in regenerative orthopedic surgeries that showed high incidences of failure secondary to large bone defects [3]. Successful bone tissue engineering products require four components: a morphogenetic signal molecule, stem cells that can deposit bony matrix upon receiving to the signal, suitable scaffolds that deliver both signal and cells to defect sites, and a well vascularized host microenvironment [4, 5]. Ideal signal molecules should be nontoxic, nonimmunogenic, and efficient in promoting the differentiation of stem cells towards osteoblasts [6].

There are two cannabinoid receptors both of which are G protein coupled receptors. Cannabinoid receptor type 1 (CB_1 _receptor) is mainly expressed in central nervous system [7], while CB_2_ receptor is predominantly present in peripheral tissue like immune system [8], liver cirrhosis [9], and atherosclerotic plaques [10]. The gene encoding CB_1_ receptor or CB_2_ receptor is usually abbreviated as CNR1 or CNR2. Traditionally, it was believed that CB_1_ receptor mediates the cannabinoid psychotropic, analgesic, and orectic effects, and CB_2_ receptor plays a role in the regulation of liver fibrosis and atherosclerosis. In more recent studies, it was shown that CB_2_ receptor deficient mice had dramatic bone loss and cortical expansion [11]. It was also indicated that a CB_2_ receptor specific agonist HU-308 enhances endocortical osteoblast number and activity and restrains trabecular osteoclastogenesis. These results demonstrate that the activation of CB_2_ signaling is essential for the maintenance of normal bone mass. Manipulating CB_2_ signaling may offer a molecular tool for the increasing osteogenic differentiation of stem cells.

In this study, we hypothesized that activation of CB_2_ receptor by chemical agonist could enhance the osteogenic differentiation and mineralization of bone marrow mesenchymal stem cells (BM-MSCs). Alkaline phosphatase activity staining and real-time PCR were performed to test the osteogenic differentiation. Alizarin red staining was carried out to examine the mineralization of BM-MSCs. Small interference RNA was used to study the role of CB_2_ receptor in osteogenic differentiation of BM-MSCs.

## 2. Materials and Methods

### 2.1. Biopsies, Cell Culture, and Expansion

The use of human material in this study has been approved by a Local Medical Ethical Committee of China Medical University. Bone marrow biopsies were obtained from patients who underwent bone marrow examinations in the First Affiliated Hospital, China Medical University, by bone marrow aspiration. Healthy donors were defined as individuals without osteoporosis. Mesenchymal stem cells (MSCs) were derived from bone marrow of healthy donors as described previously [12]. Briefly, total bone marrow was plated at a density of 50 000 cells/cm^2^ in culture flasks in MSC proliferation medium (*α*-MEM, supplemented with 10% fetal bovine serum, 1% L-glutamine, 0.2 mM ascorbic acid, 100 U/mL penicillin, 10 *μ*g/mL streptomycin, and 1 ng/mL bFGF), plus 1% heparin. Medium was refreshed every 3-4 days until confluence. Four donors of healthy and osteoporotic patients were used in this study. All reagents used for cell culture were purchased from Invitrogen (Carlsbad, CA). All chemicals were purchased from Sigma-Aldrich, unless specified.

### 2.2. Osteogenic Differentiation and CB_2_ Receptor Agonist Treatment

Osteogenic differentiation was induced by culturing MSCs in osteogenic medium (OS) containing DMEM plus 10% FBS, 0.1 nM dexamethasone, 10 mM b-glycerophosphate, 0.01 *μ*M 1,25-dihydroxy vitamin D3, and 50 *μ*M ascorbic acid in a-MEM [13]. For treatment of CB_2_ receptor agonist, UR-144 (10 uM) was added to culture medium together with OS medium from day 0 of osteogenic induction.

### 2.3. Alizarin Red Staining

After 3-week induction and treatment of CB_2_ receptor agonist UR-144 (10 nM), MSCs were fixed with 10% formalin. Then, mineralized nodules were stained with alizarin red S. After rinsing in phosphate-buffered saline (PBS), cells were incubated with 40 mM of alizarin red S (pH 4.2) for 10 min on under agitation. Cells were rinsed 5 times with water followed by 15 min washing with PBS to reduce nonspecific staining of alizarin red S. The stained nodules were observed through phase contrast microscope.

### 2.4. Alkaline Phosphatase Activity Staining

Cytochemical analysis with 5-bromo-4-chloro-3-indolyl phosphate (BCIP) and nitro blue tetrazolium chloride (NBT) was used for the staining of alkaline phosphatase. MSCs were first fixed in 10% formalin. Then cells were incubated with 300–400 *μ*L BCIP/NBT premixed solution (Sigma Aldrich, St. Luis, MO) for 8–10 min at room temperature. Cells were rinsed with water, dried, and examined with phase contract microscopy.

### 2.5. Image Quantification with ImageJ

ImageJ software was used for quantification of positively stained area. Briefly, we manually set a threshold to avoid artifacts. Then colored images were transformed into binary images. Area of positive staining was divided by total area to make percentage of positively stained area. An average was made from three technical replicates for each donor. Values represent the mean ± standard deviation of 4 donors.

### 2.6. RNA Isolation and Quantitative PCR

Samples of total RNA from chondrocytes seeded in cell culture plates or from freshly aspirated bone marrow were isolated with the QIAamp DNA Mini Kit (Qiagen, Hilden, Germany). One microgram of total RNA was reverse-transcribed into cDNA using the iScript cDNA Synthesis kit (Bio-Rad, Hercules, CA). The cDNA samples were amplified with a Pfu PCR kit (Tiangen, Beijing, China), and the specific primers were displayed in Table 1. All PCR products were resolved on a 2% agarose gel.

Real-time PCR was performed on cDNA samples by using the iQ SYBR Green Supermix (Bio-Rad, Hercules, CA). PCR reactions were carried out on MyiQ2 Two-Color Real-Time PCR Detection System (Bio-Rad, Hercules, CA) under the following conditions: cDNA was preheated for 15 min at 95°C, denatured for 5 min at 95°C, followed by 45 cycles, consisting of 15 s at 95°C, 15 s at 60°C, and 30 s at 72°C. For each reaction a melting curve was generated to test primer dimer formation and nonspecific priming. The primers for real-time PCR are listed in Table 1. Calculation of relative expression was performed with Bio-Rad iQ5 optical system software (version 2.0) using the double delta Ct method [14]. GAPDH was used for normalization.

### 2.7. Knockdown of CNR2 by Small Interference RNA (siRNA) in MSCs

Small interference RNA constructs which specifically knock down the expression of CNR2 were designed, synthesized, and cloned into a lentiviral vector (pLVshRNA-eGFP) also expressing GFP (Inovogen, Beijing, China). Details of lentiviral vector are available on the website of Inovogen (http://www.inovogen.com/lentivirus/lentivirusvector/pLVshRNA-EGFP/). Lentiviral supernatants containing siRNA constructs were packaged by the same company (Inovogen, Beijing, China) as outsource service, using triple transfection of HEK293T cells. MSCs were infected by adding viral supernatants to the culture medium. Stably infected cells were sorted by FACS based on expression of GFP (green fluorescent protein) on day 7 after infection and expanded for several passages before osteogenic differentiation.

### 2.8. Immunofluorescent Staining

MSCs with or without viral transduction were plated on glass cover slips in six-well plates 24 hours before staining. Cells were washed briefly with PBS, fixed in 4% paraformaldehyde for 30 min, at room temperature, and then permeabilized and blocked in 1% Triton-X 100 and 1% bovine serum albumin (BSA) for 15 min at room temperature. Slips were subsequently incubated overnight at 4°C with rabbit polyclonal antibodies against CB_2_ receptor (ab3561, AbCam, Cambridge, MA) or phospho-p38 MAPK (phospho T180, ab178867, AbCam, Cambridge, MA). Sequentially, slides were incubated with secondary antibodies conjugated to Alexa 594 (Invitrogen, Carlsbad, CA) or conjugated with FITC (Pierce, Rockford, IL) and nuclei were stained with 4,6-diamidino-2-phenylindole (DAPI; Molecular Probes, Eugene, OR). After rinsing with PBS, cells were examined and imaged with DMi 6000 B fluorescent microscope (Leica, Bensheim, Germany).

### 2.9. Immunohistochemical Staining

Bone marrow tissues obtained from bone marrow examination were fixed in 10% formalin and then embedded in paraffin with routine histological procedures. 5 *μ*m sections were cut for immunocytochemical staining. Sections were incubated with 3% hydrogen peroxide in methanol for 30 min to inhibit endogenous peroxidase activities. Washed with PBS, they were then blocked in 1% bovine serum albumin and 1.5% normal goat serum at room temperature for 30 min. Slides were subsequently incubated overnight at 4°C with rabbit polyclonal antibodies against CNR2 (AbCam, Cambridge, MA). Sequentially, slides were incubated with secondary biotinylated antibodies and horseradish peroxidase-conjugated streptavidin to detect the primary antibodies. The peroxidase reaction was developed using 3,3-diaminobenzidine tetrahydrochloride as chromogens. After rinsing in distilled water, slides were dehydrated in ethanol solutions, cleared in xylene, and mounted with cover slips for microscopic examination.

### 2.10. Statistical Analysis

All statistical analysis was made by using Student's *t*-test for paired samples. *P* values of <0.05 were considered as statistically significant.

## 3. Results

### 3.1. Cannabinoid Receptor 2 but Not Cannabinoid Receptor 1 Is Expressed in Bone Marrow Mesenchymal Stem Cells

**Figure 1:**
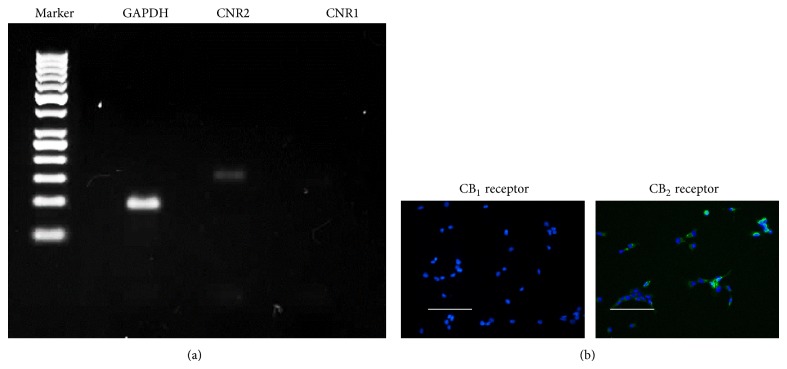
Expression of cannabinoid receptors 1 and 2 in BM-MSCs. (a) RT-PCR analysis of cannabinoid receptors (CNR) 1 and 2 genes in BM-MSCs. Expression of cannabinoid receptor 2 was confirmed in BM-MSCs. GAPDH was used as internal control. PCR products were resolved on 2% agarose gel. (b) Immunofluorescent staining was performed to detect expression of CNR1 and CNR2 in BM-MSC at protein level. Bar = 100 *μ*m.

It is believed that CB_1_ receptor is mainly expressed in central nerve system [15], while CB_2_ receptor is mainly expressed on T cells of the immune system, on macrophages and B cells, and in hematopoietic cells [16]. To test the expressions of CB_1_ and CB_2_ receptors in BM-MSCs, RT-PCR was performed with primers of the genes of two receptors.  Figure 1(a) shows that only CNR2 (the gene encoding CB_2_ receptor) is expressed in BM-MSCs. Results of immunofluorescent staining confirmed that only CB_2_ receptor is found in BM-MSCs (Figure 1(b)). CNR1 is not expressed in BM-MSCs at mRNA or protein levels.

### 3.2. Activation of Cannabinoid Receptor 2 Enhances Osteogenic Differentiation of Bone Marrow Mesenchymal Stem Cells

**Figure 2:**
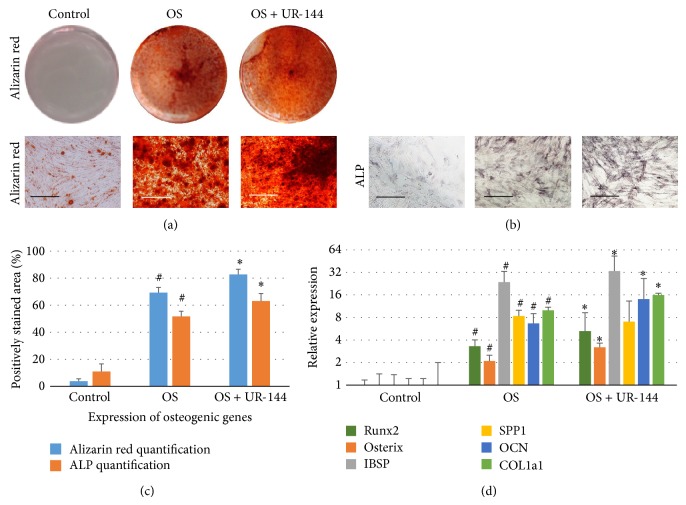
Osteogenic differentiation of BM-MSC was enhanced by agonist of cannabinoid receptor 2. (a) Alizarin red staining of BM-MSC after 3-week culture. Bar = 100 *μ*m. (b) Alkaline phosphatase staining (ALP) after 2-week culture. Bar = 100 *μ*m. (c) Quantification of positively stained area for alizarin red staining and ALP staining reveals that OS + UR-144 group has more mineralization and more ALP activity than OS group. (d) Real-time PCR was performed to analyze osteogenic genes in BM-MSC after 3-week culture (*N* = 4). GAPDH was amplified for normalization. OS = osteogenic medium. # represents significant difference when comparing OS group with control group. ∗ represents significant difference when comparing OS + UR-144 with OS group.

To study the role of cannabinoid signaling in mineralization, specific agonist for CB_2_ receptor UR-144 is used to activate CB_2_ receptor on BM-MSCs during osteogenic differentiation. Alizarin red staining was performed to examine the mineralized nodules formed by BM-MSC cultured in osteogenic medium after 3 weeks. BM-MSCs cultured in osteogenic medium plus 10 nM of UR-144 show stronger staining than cells culture in osteogenic medium only (Figure 2(a)). Alkaline phosphatase activity staining carried out at week 2 after induction confirmed that CB_2_ receptor activation by agonist enhances ALP activity (Figure 2(b)). Quantification of mineralization related genes by real-time PCR at week 3 of induction also indicated that CB_2_ receptor activation by agonist increases the expression levels of genes regulating osteogenic differentiation (Figure 2(c)). These genes include Runx2, Osterix, IBSP, SPP1, OCN, and COL1a1.

### 3.3. Inhibition of Cannabinoid Receptor 2 by siRNA Reduces Osteogenic Differentiation of Bone Marrow Mesenchymal Stem Cells

**Figure 3:**
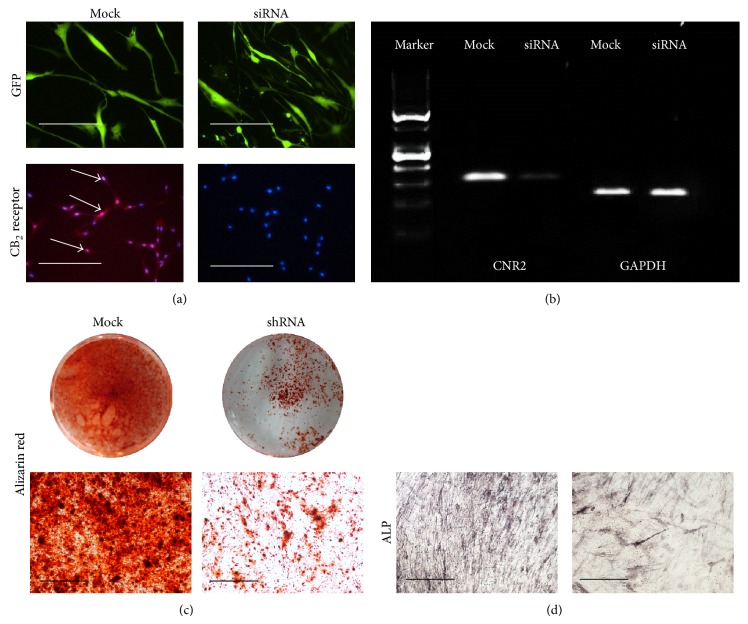
Osteogenic differentiation of BM-MSC was inhibited by knockdown of cannabinoid receptor 2. (a) BMSCs infected with virus containing shRNA sequence against cannabinoid receptor 2 (CNR2) or mock sequence. Green fluorescent protein (GFP) marks successfully infected cells. Bar = 100 *μ*m. Immunofluorescent staining was performed to examine the expression of CB_2_ receptor on infected cells. White arrows indicate positive staining for CB_2_ receptor. (b) RT-PCR analysis of cannabinoid receptors (CNR) 1 and 2 genes in BM-MSC. Expression of cannabinoid receptor 2 was confirmed in BM-MSCs. GAPDH was used as internal control. PCR products were resolved on 2% agarose gel. (c) Alizarin red staining of BM-MSC after 3-week culture. Bar = 100 *μ*m. (d) Alkaline phosphatase staining (ALP) after 2-week culture. Bar = 100 *μ*m.

To test if inhibition of CB_2_ receptor would influence the mineralization of BM-MSCs, small interference RNA (siRNA) technology was used to knock down the expression of CB_2_ receptor in BM-MSCs. Constructs of siRNA were introduced into BM-MSCs by lentivirus. Green fluorescent protein (GFP) was applied as labels of positive transduction. After FACS sorting, all cells were positive for GFP (Figure 3(a)). Meanwhile, immunofluorescent staining and RT-PCR were performed to test the efficiency of knockdown. As shown in Figures 3(a) and 3(b), CB_2_ receptor is present in BM-MSCs infected with mock sequence but is absent in BM-MSCs infected with siRNA sequence. BM-MSCs infected with either mock or siRNA sequence were cultured in osteogenic medium for mineralization assay. Alizarin red S staining performed at week 3 indicated that knockdown of CNR2 gene significantly reduced the accumulation of calcium in extracellular matrix (Figure 3(c)). Alkaline phosphatase at week 2 staining also showed that absence of CB_2_ receptor inhibited the activity of alkaline phosphatase in BM-MSCs.

### 3.4. Effects of Cannabinoid Receptor 2 Signaling on Mineralization Are Mediated through p38 Mitogen-Activated Protein Kinase

**Figure 4:**
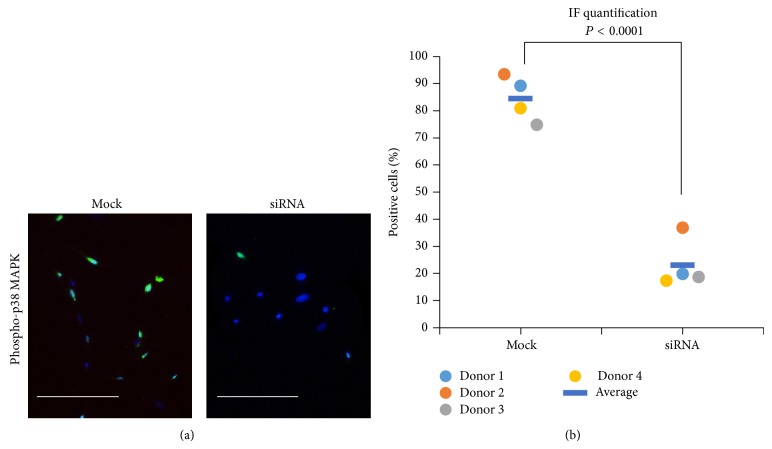
Knockdown of cannabinoid receptor 2 reduces phosphorylation of p38 MAPK. (a) Expression of phosphorylated p38 MAPK (phosphor-p38 MAPK) in BMSCs infected with virus containing mock sequence or siRNA sequence against cannabinoid receptor 2 (CNR2). Bar = 100 *μ*m. (b) Quantification of immunofluorescent (IF) images shows that less siRNA infected BM-MSCs are positive for phosphor-p38 MAPK than mock infected BM-MSCs (*N* = 4).

Since activation of p38 mitogen-activated protein kinase (p38 MAPK) had been shown to stimulate osteogenic differentiation, we hypothesized that effects of CB_2_ receptor signaling on mineralization could be mediated through p38 MAPK.  Figure 4(a) shows phosphorylation of p38 MAPK in BMSCs infected with virus containing mock sequence or siRNA sequence against CNR2. Quantification of immunofluorescent (IF) images confirmed the impression that phosphorylation of p38 MAPK BM-MSCs infected with siRNA sequences is much less than that in BM-MSCs infected with mock sequences (*P* < 0.001). Percentage of positively stained cells drops from about 80% (in mock) to roughly 20% (in siRNA).

### 3.5. Cannabinoid Receptor 2 Is Less Expressed in Bone Marrow of Osteoporotic Patients Than in Bone Marrow of Healthy Donor

**Figure 5:**
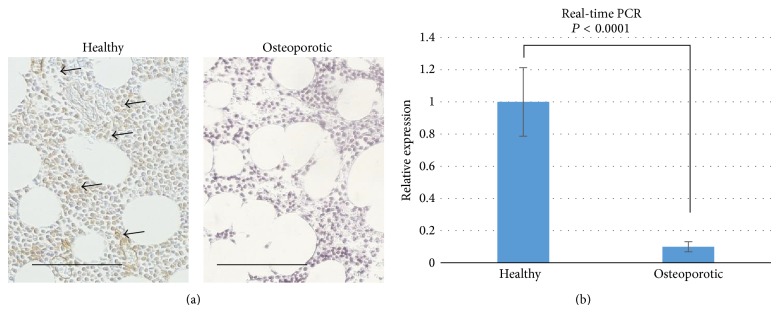
Expression of cannabinoid receptor 2 in healthy and osteoporotic patients. (a) Immunohistochemistry shows presence of CB_2_ receptor in the bone marrow tissue of healthy donor, and absence in osteoporotic patients. Bar = 100 *μ*m. Arrowheads indicate the positively stained cells. (b) Real-time PCR analysis of CNR2 bone morrow tissue of healthy donor and osteoporotic donor (*N* = 4).

Finally, the expression of CB_2_ receptor was tested in human bone marrow tissue. Results of immunohistochemistry indicated that CB_2_ receptor is abundant in the bone marrow tissue of healthy donor. Meanwhile, barely any positive cells can be found in bone marrow of osteoporotic patients (Figure 5(a)). Total RNA of bone marrow tissue from both healthy and osteoporotic donors were isolated for real-time PCR analysis. CNR2 expression in osteoporotic bone marrow is only 10% of that in healthy bone marrow.

## 4. Discussion

In this study, activation of CB_2_ receptor was shown to enhance the osteogenic differentiation and mineralization of BM-MSCs. Cells treated with CB_2_ receptor agonist presented higher alkaline phosphatase activity staining, more expression of osteogenic genes, and more deposition of calcium in extracellular matrix. Knockdown of CB_2_ receptor by small interference RNA in BM-MSCs severely reduced the osteogenic differentiation of BM-MSCs. Our data also indicated that activation of CB_2_ receptor was very likely acting through phosphorylation of p38 MAPK.

MSCs are considered as multipotent stem cells that can be isolated from many adult tissues, including bone marrow, dermis, muscles, ligament and placenta, and fat tissue [17]. MSCs may be expanded* in vitro* for many passages while keeping their potential of differentiating into multilineages of tissues [18]. Bone morrow MSCs are derived from the nonhaematopoietic portion of the bone marrow [19]. It is believed that MSCs are an attractive cell source for bone tissue engineering [20]. In this study, BM-MSCs are shown to express CB_2_ receptor and be responsive to its agonist. CB_2_ receptor agonist was shown to be useful in promoting the performance of BM-MSCs in bone tissue engineering. For example, as small molecules, UR-144 can be easily integrated into scaffolds or loaded on nanoparticle as controlled released drugs.

The endogenous cannabinoids can bind to both CB_1_ and CB_2_ receptors. Both receptors contain seven-transmembrane domain. The two receptors are coupled to a subclass of G proteins that inhibit guanine nucleotide-binding and adenylyl cyclase activity [21]. Even though they have 44% identity in amino acids sequences, CB_1_ and CB_2_ are functionally different. One example is the selective regulation of ion channels by only CB_1_ receptors [22]. Regarding bone mass regulation and bone turnover, CB_1_ and CB_2_ receptors also play distinctive roles. Inactivation of CB_1_ receptor could promote bone mass and prevent osteoporotic-like bone loss induced by ovariectomy [23]. On the other hand, CB_2_ receptor could regulate osteoclast formation and contributes to ovariectomy-induced bone loss. Our results support that activation of CB_2_ receptors increases bone formation by inducing BM-MSCs differentiation.

Interestingly, knockdown of CB_2_ receptor reduced the ALP activity and calcium accumulation. This result implies that the CB_2 _might play an essential role in the differentiation steps of BM-MSCs towards osteoblasts. This also suggested that MSCs might produce endogenous cannabinoid to allow themselves to differentiate into osteogenic lineage. The autocrine effects of cannabinoid of MSCs would need more investigation in the future.

Taken together, our data demonstrated a new mechanism in which osteogenic differentiation and mineralization can be enhanced by activating CB_2_ receptor. Our results also suggested that lack of CB_2_ receptor is associated with osteoporosis. Increasing CB_2_ signaling may be useful in both prompting bone tissue engineering products and treating osteoporotic patients.

## Figures and Tables

**Table 1:** Sequences for primers.

Gene name	NCBI gene ID	Sequence (5′ → 3′)	Length of amplicon
Cannabinoid receptor 1 (CNR1)	1268	Forward: GTGTTCCACCGCAAAGATAGC Reverse: GGGGCCTGTGAATGGATATGT	130

Cannabinoid receptor 2 (CNR2)	1269	Forward: AGCCCTCATACCTGTTCATTGG Reverse: GTGAAGGTCATAGTCACGCTG	154

Runt-related transcription factor 2 (RUNX2)	860	Forward: TGGTTACTGTCATGGCGGGTA Reverse: TCTCAGATCGTTGAACCTTGCTA	101

Osterix (OSX)		Forward: CCTCTGCGGGACTCAACAAC Reverse: AGCCCATTAGTGCTTGTAAAGG	128

Integrin-binding sialoprotein (IBSP)	3381	Forward: CACTGGAGCCAATGCAGAAGA Reserve: TGGTGGGGTTGTAGGTTCAAA	106

Osteocalcin (OCN)	632	Forward: CACTCCTCGCCCTATTGGC Reserve: CCCTCCTGCTTGGACACAAAG	112

Secreted phosphoprotein 1 (SPP1)	6696	Forward: GAAGTTTCGCAGACCTGACAT Reserve: GTATGCACCATTCAACTCCTCG	91

WNT5A	7474	Forward: ATTCTTGGTGGTCGCTAGGTA Reverse: CGCCTTCTCCGATGTACTGC	159

Glyceraldehyde-3-phosphate dehydrogenase (GAPDH)	2597	Forward: CTGGGCTACACTGAGCACC Reserve: AAGTGGTCGTTGAGGGCAATG	101

## References

[1] Burg K. J. L., Porter S., Kellam J. F. (2000). Biomaterial developments for bone tissue engineering. *Biomaterials*.

[2] Grayson W. L., Fröhlich M., Yeager K. (2010). Engineering anatomically shaped human bone grafts. *Proceedings of the National Academy of Sciences of the United States of America*.

[3] Dekker R. J., De Bruijn J. D., Van Den Brink I., Bovell Y. P., Layrolle P., Van Blitterswijk C. A. (1998). Bone tissue engineering on calcium phosphate-coated titanium plates utilizing cultured rat bone marrow cells: a preliminary study. *Journal of Materials Science: Materials in Medicine*.

[4] Frak V., Croteau I., Bourbonnais D., Duval C., Duclos C., Cohen H. (2007). Simulation modifies prehension: evidence for a conjoined representation of the graspable features of an object and the action of grasping it. *PLoS ONE*.

[5] Lin Y., Tang W., Wu L. (2008). Bone regeneration by BMP-2 enhanced adipose stem cells loading on alginate gel. *Histochemistry and Cell Biology*.

[6] Salgado A. J., Coutinho O. P., Reis R. L. (2004). Bone tissue engineering: state of the art and future trends. *Macromolecular Bioscience*.

[7] Zimmer A., Zimmer A. M., Hohmann A. G., Herkenham M., Bonner T. I. (1999). Increased mortality, hypoactivity, and hypoalgesia in cannabinoid CB1 receptor knockout mice. *Proceedings of the National Academy of Sciences of the United States of America*.

[8] Munro S., Thomas K. L., Abu-Shaar M. (1993). Molecular characterization of a peripheral receptor for cannabinoids. *Nature*.

[9] Julien B., Grenard P., Teixeira-Clerc F. (2005). Antifibrogenic role of the cannabinoid receptor CB2 in the liver. *Gastroenterology*.

[10] Steffens S., Veillard N. R., Arnaud C. (2005). Low dose oral cannabinoid therapy reduces progression of atherosclerosis in mice. *Nature*.

[11] Ofek O., Karsak M., Leclerc N. (2006). Peripheral cannabinoid receptor, CB2, regulates bone mass. *Proceedings of the National Academy of Sciences of the United States of America*.

[12] Fernandes H., Dechering K., van Someren E. (2009). The role of collagen crosslinking in differentiation of human mesenchymal stem cells and MC3T3-E1 cells. *Tissue Engineering—Part A*.

[13] Wu L., Wu Y., Lin Y. (2007). Osteogenic differentiation of adipose derived stem cells promoted by overexpression of osterix. *Molecular and Cellular Biochemistry*.

[14] Livak K. J., Schmittgen T. D. (2001). Analysis of relative gene expression data using real-time quantitative PCR and the 2^−ΔΔ*C*_T_^ method. *Methods*.

[15] Lazenka M. F., Selley D. E., Sim-Selley L. J. (2013). Brain regional differences in CB1 receptor adaptation and regulation of transcription. *Life Sciences*.

[16] Basu S., Dittel B. N. (2011). Unraveling the complexities of cannabinoid receptor 2 (CB2) immune regulation in health and disease. *Immunologic Research*.

[17] Caplan A. I. (1991). Mesenchymal stem cells. *Journal of Orthopaedic Research*.

[18] Le Blanc K., Pittenger M. F. (2005). Mesenchymal stem cells: progress toward promise. *Cytotherapy*.

[19] Gronthos S., Zannettino A. C. W., Hay S. J. (2003). Molecular and cellular characterisation of highly purified stromal stem cells derived from human bone marrow. *Journal of Cell Science*.

[20] Conrad C., Huss R. (2005). Adult stem cell lines in regenerative medicine and reconstructive surgery. *Journal of Surgical Research*.

[21] Rhee M.-H., Vogel Z., Barg J. (1997). Cannabinol derivatives: binding to cannabinoid receptors and inhibition of adenylylcyclase. *Journal of Medicinal Chemistry*.

[22] Felder C. C., Joyce K. E., Briley E. M. (1995). Comparison of the pharmacology and signal transduction of the human cannabinoid CB1 and CB2 receptors. *Molecular Pharmacology*.

[23] Idris A. I., Van't Hof R. J., Greig I. R. (2005). Regulation of bone mass, bone loss and osteoclast activity by cannabinoid receptors. *Nature Medicine*.

